# Choroidal vascularity across aging and the spectrum of age-related macular degeneration: an AI-based OCT study

**DOI:** 10.1007/s00417-026-07269-2

**Published:** 2026-04-30

**Authors:** Benja Vahldiek, Lukas Heine, Anna Vahldiek, Jasper Schröter, Jan-Niklas Wolf, Michael Swora, Lars Reissberg, Laurenz Pauleikhoff, Jens Kleesiek, Daniel Pauleikhoff

**Affiliations:** 1 Institute for AI in Medicine, University Medicine Essen, Essen, North-Rhine Westfalia Germany; 2 Institute for AMD Research, Münster, Germany; 3 Department of Ophthalmology, University Hospital Hamburg-Eppendorf, Hamburg, Germany

**Keywords:** Age-related macular degeneration, Choroidal morphology, OCT, AI analysis, CVI

## Abstract

**Purpose:**

To characterize the choroidal morphology across a spectrum of participants affected by normal aging as well as age-related macular degeneration (AMD) using various metrics.

**Methods:**

Four cohorts were analyzed: healthy young (*n* = 42), healthy elderly (*n* = 19), iAMD (*n* = 20), and treatment-naive nAMD eyes (*n* = 79). Quantification of choroidal volume (CV) and choroidal vessel volume (VV) resulting in choroidal vascularity index (CVI) as well as mean choroidal thickness (CT), vessel thickness (VT) and foveal vessel area (FVA) and vessel count (FVC) were derived from spectral-domain optical coherence tomography using an artificial intelligence (AI)-based semantic segmentation model with manual validation. Cohort differences were statistically assessed.

**Results:**

All choroidal parameters were reduced in healthy elderly, iAMD and nAMD eyes compared with young controls. While most of these reduced choroidal measurements were largely similar between healthy elderly, iAMD and nAMD eyes, only CVI and FVA showed a specific reduction in nAMD eyes.

**Conclusions:**

This study demonstrates a decline in several choroidal parameters with age, which can be quantified using AI-based segmentations. While age-related reductions were comparable between elderly and iAMD eyes, a significantly higher reduction of the CVI and FVA was observed in eyes with nAMD which may indicate a more pronounced alteration of choroidal structure in this condition.

## Abstract (fr)

**Key messages:**

*****What is known***:**

- Choroidal morphology changes with age and can be quantified using parameters such as thickness, volume, and the choroidal vascularity index.

*****What is new***:**

- Using AI-based segmentations and quantifications of various morphological choroidal parameters, a substantial decrease of choroidal characteristics is attributable to age.
- Age-related choroidal changes are comparable in healthy elderly and intermediate AMD eyes, whereas neovascular AMD shows afurther reduction of choroidal vasculature.

## Introduction

Age-related macular degeneration (AMD) is a leading cause of irreversible vision loss in aging populations and represents a major public health burden [1, 2]. Despite advances in imaging and therapy, its pathophysiology remains incompletely understood, particularly regarding the relative contribution of retinal pigment epithelium (RPE), photoreceptor dysfunction and choroidal vascular alterations [1, 3, 4].

Neovascular AMD (nAMD) is characterized by the development of macular neovascularization (MNV), leading to exudation, hemorrhage, and rapid visual deterioration [5]. Anti-vascular endothelial growth factor (Anti-VEGF) therapy has improved short-term outcomes by controlling exudation and stabilizing visual function [6]. However, long-term treatment is usually required, progressive visual decline is common and has been associated with the development and progression of macular atrophy [7–10]. Thus, structures beyond the neurosensory retina, particularly the choroid, may represent critical therapeutic targets.

The choroid plays a central role in retinal homeostasis as the principal vascular supply of the outer retina. It delivers oxygen and nutrients to the RPE and photoreceptors, supporting their high metabolic demand [4]. Impairment of choroidal perfusion may result in tissue hypoxia and metabolic imbalance, oxidative stress and accumulation of metabolic waste products [3, 4, 11]. Hypoxia-driven signaling pathways may further promote VEGF expression and MNV formation as a compensatory response, linking the possibility of choroidal dysfunction to the development of nAMD [12].

Aging is associated with choroidal thinning and vascular rarefaction [4, 13]. Beyond these age-related alterations, disease-specific changes across different stages of AMD were observed. In intermediate AMD (iAMD) and geographic atrophy (GA), progressive impairment of the choriocapillaris has been described, along with regional thinning of the outer choroid [14, 15]. In nAMD, enlargement and structural alteration of vessels in Haller’s and Sattler’s layers in treatment-naive nAMD were described [16, 17].

However, choroidal thickness (CT) alone provides limited insight into vascular integrity and does not differentiate between luminal and stromal components [4, 14, 18]. The choroidal vascularity index (CVI), defined as the ratio of luminal to total choroidal area, has emerged as a robust parameter for assessing choroidal vascular structure, which has been shown to be less dependent on physiological factors such as axial length, diurnal variation and systemic influences than CT [19, 20].

Although automated approaches for choroidal and vascular segmentation are increasingly available, many studies have focused on binarization methods quantify the choroidal vasculature by applying intensity-based thresholding to individual single B-scans or region-averaged measurements [18, 19, 21, 22]. In these approaches, OCT B-scan images are thresholded using local algorithms (e.g., the modified Niblack method) to separate dark luminal pixels from brighter stromal tissue. As these methods are typically applied to individual B-scans or limited sampling regions, fully volumetric analyses of choroidal morphologic parameters, particularly in the context of aging and different AMD phenotypes, remain limited. In contrast, AI-based three-dimensional segmentation analyzes the entire OCT volume and directly identifies choroidal vessels at the voxel level. This enables reconstruction and quantitative assessment of the vascular network in three dimensions and allows anatomy-aware quantification of choroidal vascular structure. The aim of the present study was to characterize age- and AMD-related patterns of choroidal remodeling using three-dimensional, AI-based annotation and quantification of choroidal and vascular parameters including CT and CVI.

These parameters were compared between young and elderly healthy eyes, as well as between elderly healthy eyes and eyes with iAMD or nAMD. This approach may help to elucidate age- and AMD-related choroidal alterations and to provide new insights into the potential role of the choroid in AMD pathogenesis.

## Methods

### Study design and participants

This was a cross-sectional observational study with prospective and retrospective cohorts designed to compare choroidal morphology across healthy eyes and different stages of AMD, without longitudinal follow-up.

Data for the healthy elderly, iAMD, and treatment-naive nAMD cohorts were retrospectively obtained from an observational treatment study conducted by AMD-Netz Münster [23]. Since this dataset did not include a healthy young control cohort, an additional group of healthy young individuals was prospectively recruited at the Institute for AI in Medicine, University Medicine Essen. This cohort served as a reference group to distinguish physiological age-related choroidal changes from disease-related alterations associated with AMD. The study included anonymized data evaluation.

### Diagnosis

The AMD diagnoses were verified by an experienced retinal specialist (D.P.) with multimodal imaging, including fluorescein angiography using a Heidelberg Retina Angiograph 2 (HRA2) and structural OCT using the Heidelberg Spectralis system (Heidelberg Engineering).

### Study cohorts

A total of 160 eyes of 149 individuals were included and divided into four cohorts: healthy young, healthy elderly, iAMD and nAMD.

**Fig. 1:**
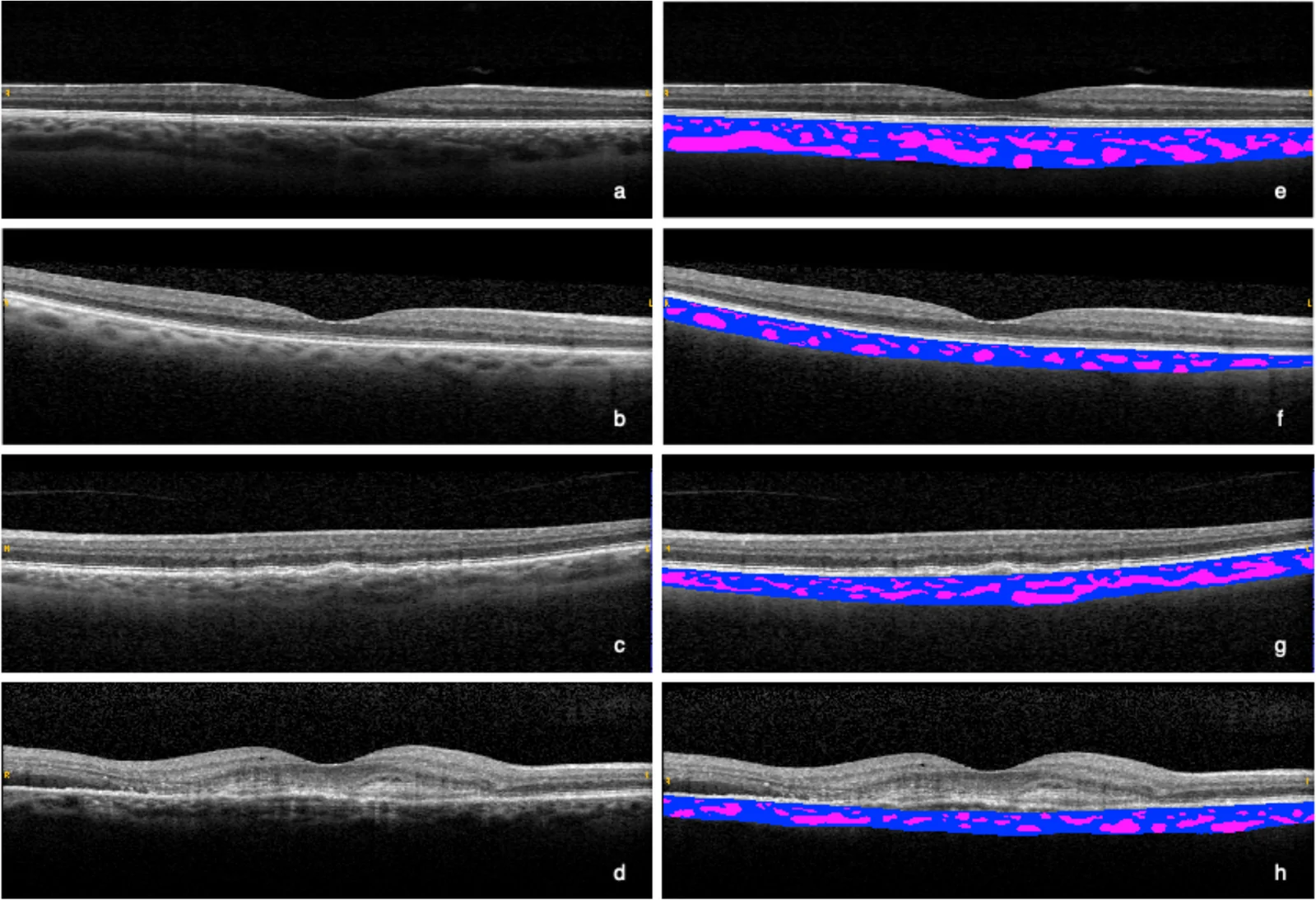
Representative OCT B-scan and annotated choroid of a healthy young (**a**, **e**), healthy elderly (**b**, **f**), iAMD (**c**, **g**) and nAMD (**d**, **h**) eyes. Each scan is shown both without annotation (**a**–**d**) and with artificial intelligence-based annotations (**e**–**h**) of choroidal vessels (pink) and choroidal stroma (blue)

The healthy young cohort consisted of individuals with physiological macular morphology, best-corrected visual acuity ≥ 20/32 and no history of retinal disease, intraocular treatment, or systemic vascular disease (Fig. 1a, e).

The healthy elderly cohort showed no signs of AMD or other retinal pathology on structural OCT imaging (Fig. 1b, f).

Eyes with iAMD were characterized by the presence of drusen as small dome-shaped elevations of hyperreflective material under the RPE, without evidence of GA or MNV.

Eyes with treatment-naive nAMD characterized by MNV were included (Fig. 1d, h).

In these elderly cohorts no information about potential systemic vascular disease was available.

### Imaging acquisition

 Macular optical coherence tomography (OCT) volumes were acquired using the Heidelberg Spectralis system (Heidelberg Engineering, Heidelberg, Germany) as 6 × 6 mm macular cube scans centered on the fovea with a depth of approximately 2 mm. Each volume comprised at least 49 B-scans. Automatic real-time averaging of more than 12 frames was applied to improve signal quality, and automated foveal alignment was used to ensure consistent scan positioning.

### Image analysis

#### Semantic choroidal segmentation

Choroidal and vascular masks were obtained using a previously developed and validated semantic segmentation model by Heine et al., which has been successfully applied to nAMD imaging analyses [24, 25].

In addition to the previously annotated structures (retinal layers and choroid), choroidal vessels were manually annotated by trained graders with domain-specific expertise to generate a dedicated ground truth dataset. Based on these annotations, a segmentation model was trained to predict voxel-wise binary masks of choroidal vessels in unseen OCT volumes. All predicted masks were subsequently visually inspected and, if necessary, minimally corrected by trained graders to ensure high-quality segmentation while maintaining scalability across the dataset (Fig. 1).

#### Quantified choroidal parameters

The parameters chosen to quantify choroidal morphology were mean CT [mm] and mean vessel thickness (VT [mm]) as well as choroidal volume (CV [mm^3^]) and choroidal vessel volume (VV [mm^3^]) resulting in the choroidal vascularity index (CVI [%] = VV/CV * 100) and the foveal vessel area (FVA [mm^2^]) and foveal vessel count (FVC).

Mean vessel thickness (VT) was derived from the voxel-wise choroidal vessel segmentations. For each A-scan (i.e., each axial voxel column along the depth direction), all voxels classified as vessel were identified. If vessel voxels were present, vessel thickness was defined as the sum of all vessel voxels along that column. Voxel counts were converted into physical units using the voxel spacing provided in the scan metadata. This procedure was repeated across all A-scans within the OCT volume, and the mean vessel thickness was calculated as the average across all A-scans containing vessel voxels.

### Statistical analysis

**Fig. 2:**
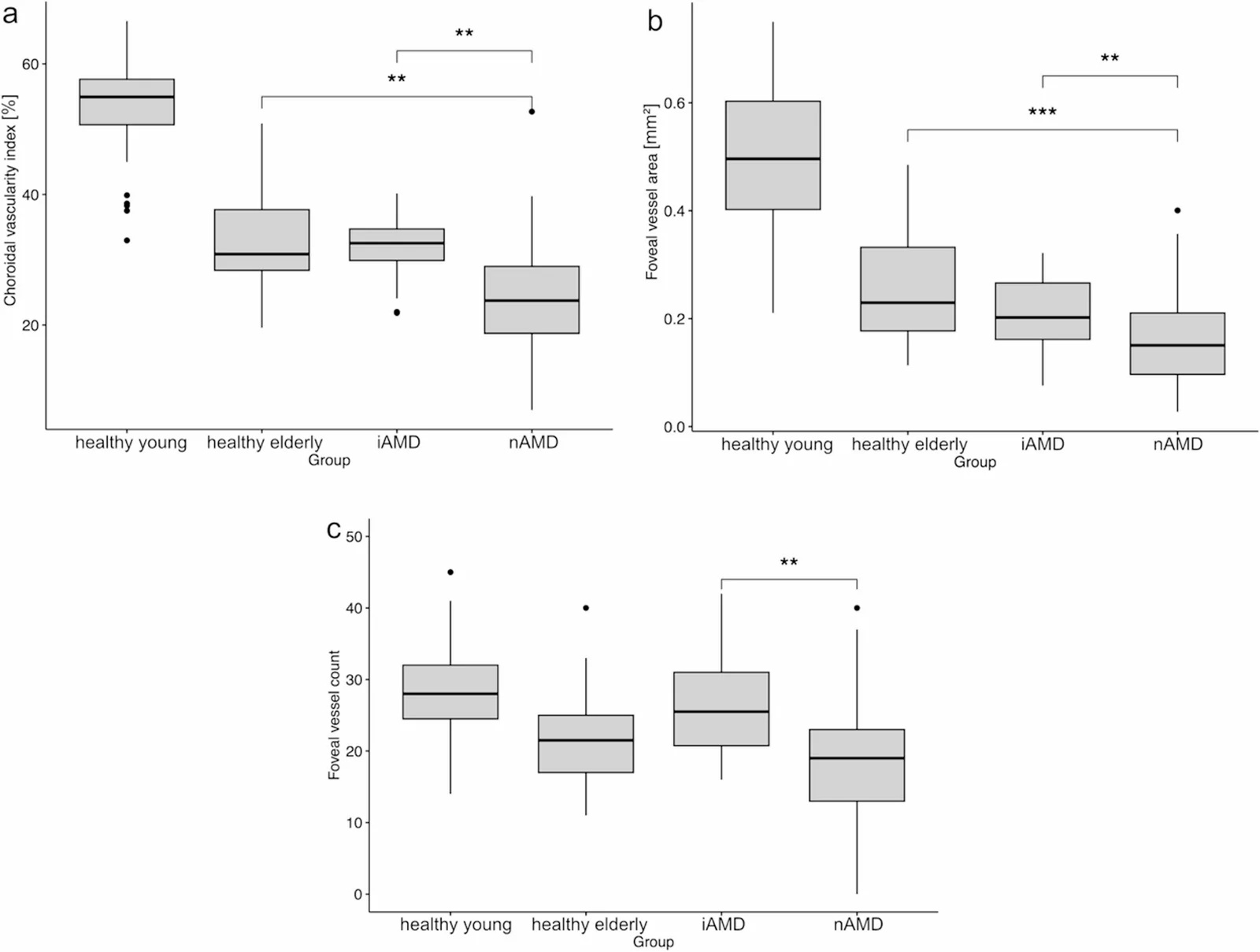
Comparison between four study cohorts (healthy elderly as reference) of the choroidal vascular index (CVI [%] = VV/CV * 100) (**a**), foveal vessel area [mm²] (**b**) and foveal vessel count (**c**). Differences in-between groups are shown using brackets, with significant p-values denoted using asterisks (* p = < 0.05, **= <0.01, *** = <0.001). Since all groups differed significantly from the young healthy cohort (except for the comparison between healthy young and iAMD for FVC), p-values for these differences are not shown

For each parameter, analysis was performed as follows: cohorts were first analyzed by means of descriptive statistical summaries. Due to small to medium unequal sample sizes within cohorts, normality and presence of outliers was assessed via Boxplots stratified by cohort and by deriving diagnostic plots in form of a QQ- and Scale-Location plot based on an ANOVA. For statistical significance testing, a nonparametric rank-based analysis was conducted by first performing a Kruskal-Wallis test. In case its p-value fell below 0.05, meaning the null hypothesis of no differences between any groups could be rejected, pairwise two-sample Wilcoxon tests were carried out and a Bonferroni-Holm adjustment for multiple testing was applied. No correction was applied for multiple testing across parameters due to the exploratory nature of this analysis. All statistical tests were performed two sided. The significance level was set at 0.05. Graphs for Fig. 2 were created using R Studio (Version 2025.09.2 + 418) and the ggplot2 package.

## Results

The study included 160 eyes from 149 individuals.

The healthy young cohort comprised 42 individuals (27 males, 15 females; mean age 31.1 ± 7.4 years, range 22–44 years). The healthy elderly cohort included 19 eyes (8 males, 11 females; mean age 73.0 ± 5.8 years, range 60–85 years). The iAMD cohort consisted of 20 eyes (9 males, 11 females; mean age 77.5 ± 5.7 years, range 69–88 years), and 79 treatment-naive nAMD eyes were analyzed (28 males, 51 females; mean age 75.4 ± 7.5 years, range 60–90).

All choroidal parameters were reduced in healthy elderly, iAMD and nAMD eyes compared with young controls (Table 1).

**Table 1:** Analysis of the different choroidal morphologic parameters of the four cohorts healthy young, healthy elderly, intermediate AMD, neovascular AMD

Characteristic	Healthy young (*n* = 42)	Healthy elderly (*n* = 19)	iAMD (*n* = 20)	nAMD (*n* = 79)
choroidal thickness (CT) [mm]				
mean (SD)	0.173 (0.025)	0.144 (0.022)	0.124 (0.025)	0.141 (0.029)
median (Q1, Q3)	0.180 (0.163, 0.186)	0.145 (0.128, 0.155)	0.124 (0.108, 0.138)	0.147 (0.119, 0.164)
min, max	0.090, 0.215	0.100, 0.182	0.082, 0.171	0.085, 0.226
vessel thickness (VT) [mm]				
mean (SD)	0.099 (0.020)	0.060 (0.013)	0.051 (0.011)	0.052 (0.011)
median (Q1, Q3)	0.100 (0.088, 0.114)	0.059 (0.052, 0.069)	0.052 (0.044, 0.059)	0.052 (0.046, 0.059)
min, max	0.043, 0.140	0.037, 0.088	0.026, 0.068	0.028, 0.089
choroidal volume (CV) [mm^3^]				
mean (SD)	6.462 (0.897)	4.939 (0.702)	4.076 (1.000)	4.603 (0.954)
median (Q1, Q3)	6.592 (5.945, 7.053)	5.027 (4.549, 5.242)	4.043 (3.363, 4.835)	4.588 (3.952, 5.320)
min, max	3.880, 8.112	3.544, 6.267	2.452, 6.071	2.560, 7.188
vessel volume (VV) [mm^3^]				
mean (SD)	3.480 (0.737)	1.647 (0.505)	1.307 (0.382)	1.094 (0.411)
median (Q1, Q3)	3.523 (2.964, 4.167)	1.543 (1.215, 2.020)	1.383 (1.058, 1.548)	1.109 (0.821, 1.334)
min, max	1.547, 5.029	0.824, 2.615	0.575, 1.946	0.295, 3.018
choroidal vascularity index^a^ (CVI) [%]				
mean (SD)	53.720 (7.955)	33.000 (7.630)	31.821 (5.002)	26.657 (7.696)
median (Q1, Q3)	54.933 (50.467, 57.654)	30.861 (28.018, 38.308)	33.133 (29.372, 34.755)	26.864 (22.506, 31.621)
min, max	32.964, 66.571	19.612, 50.873	21.438, 39.644	9.670, 54.594
foveal vessel area (FVA) [mm²]				
mean (SD)	0.499 (0.132)	0.255 (0.102)	0.211 (0.068)	0.159 (0.082)
median (Q1, Q3)	0.496 (0.401, 0.607)	0.230 (0.176, 0.334)	0.202 (0.161, 0.267)	0.151 (0.095, 0.214)
min, max	0.211, 0.750	0.113, 0.485	0.076, 0.322	0.027, 0.401
foveal vessel count (FVC)				
mean (SD)	28.238 (6.324)	22.632 (6.318)	26.500 (7.871)	19.316 (7.410)
median (Q1, Q3)	28 (24, 32)	24 (17, 26)	25.5 (20.5, 31)	20 (13, 20)
min, max	14, 45	14, 40	16.000, 42.000	4, 40

^a^ Calculated as the ratio of vessel volume to choroidal volume (VV/CV * 100)

*CT* was highest in healthy young eyes and reduced in healthy elderly, iAMD, and nAMD eyes. *VT* followed a similar pattern, with the highest values in healthy young eyes and markedly lower values in the remaining groups.

*CV* and *VV* were greatest in healthy young eyes and reduced in healthy elderly, iAMD, and nAMD eyes, with the lowest *VV* observed in nAMD. Correspondingly, *CVI* was highest in healthy young eyes and progressively decreased across healthy elderly, iAMD, and nAMD eyes (Fig. 2A).

*FVA* was largest in healthy young eyes and reduced in all other groups, with the lowest values observed in nAMD eyes (Fig. 2B). *FVC* showed a similar trend, with the highest values in healthy young eyes and a pronounced reduction in nAMD eyes compared with healthy elderly and iAMD.

For all parameters, the global Kruskal–Wallis test was highly significant (*p* < 0.001). Subsequent pairwise Wilcoxon tests demonstrated highly significant differences between healthy young eyes and all other groups (*p* < 0.0001, except for *FVC* where *p* = 0.0052). Comparisons between all cohorts including p-values are summarized in Fig. 2, Tables 1 and 2.  

**Table 2:** Pairwise p-values for differences between the four cohorts healthy young, healthy elderly, intermediate AMD, neovascular AMD

Characteristic	Healthy elderly (*n* = 19)	iAMD (*n* = 20)	nAMD (*n* = 79)
choroidal thickness (CT) [mm]			
healthy young (*n* = 42)	< 0.001***	< 0.001***	< 0.001***
healthy elderly (*n* = 19)		0.0491*	0.9570
iAMD (*n* = 20)			0.0491*
vessel thickness (VT) [mm]			
healthy young (*n* = 42)	< 0.001***	< 0.001***	< 0.001***
healthy elderly (*n* = 19)		0.0705	0.0234*
iAMD (*n* = 20)			0.8925
choroidal volume (CV) [mm^3^]			
healthy young (*n* = 42)	< 0.001***	< 0.001***	< 0.001***
healthy elderly (*n* = 19)		0.0129*	0.1664
iAMD (*n* = 20)			0.0891
vessel volume (VV) [mm^3^]			
healthy young (*n* = 42)	< 0.001***	< 0.001***	< 0.001***
healthy elderly (*n* = 19)		0.0536	< 0.001***
iAMD (*n* = 20)			0.0497*
choroidal vascularity index^a^ (CVI) [%]			
healthy young (*n* = 42)	< 0.001***	< 0.001***	< 0.001***
healthy elderly (*n* = 19)		0.8567	0.0070**
iAMD (*n* = 20)			0.0038**
foveal vessel area (FVA) [mm²]			
healthy young (*n* = 42)	< 0.001***	< 0.001***	< 0.001***
healthy elderly (*n* = 19)		0.1937	0.0008***
iAMD (*n* = 20)			0.0099**
foveal vessel count (FVC)			
healthy young (*n* = 42)	0.0052**	0.2785	< 0.001***
healthy elderly (*n* = 19)		0.2785	0.1372
iAMD (*n* = 20)			0.0037**

* *p* < 0.05, ** *p* < 0.01, *** *p* < 0.001

^a^ Calculated as the ratio of vessel volume to choroidal volume (VV/CV * 100)

## Discussion

In the study, younger individuals exhibited a thicker and more vascularized choroid compared to older participants. This pattern was consistent across all analyzed parameters and concordant with early histological choroidal corrosion cast studies and imaging-based investigations [13, 21]. Changes were observed irrespective of the presence or absence of drusen: physiological aging alone, although associated with substantial choroidal remodeling, is not sufficient to explain AMD development.

The CVI has emerged as a robust quantitative parameter for characterizing choroidal vascular morphology compared to CT alone [18–21]. Lower observed CVI values may be attributable to methodological differences.

Many prior studies relied on binarization-based approaches that are sensitive to signal attenuation and threshold selection [18–21, 26]. In contrast, the semantic annotation-based three-dimensional segmentation applied in this study enables anatomically precise, threshold-free, morphology-aware differentiation between vascular and stromal components [24, 25]. This strategy is particularly relevant for evaluating choroidal structures in nAMD, as volumetric analysis reduces, compared with conventional B-scan binarization, the potential influence of MNV-related shadowing and therefore provides more robust and reliable results. By applying this volumetric AI-based approach across healthy aging and different AMD stages, the present study provides a comprehensive characterization of choroidal vascular alterations.

Consistent with previous investigations, CVI declined with age, reflecting progressive loss of the vascular compartment with relative stromal expansion [19, 22].

While differences in choroidal morphology between young and healthy elderly eyes were observed, most metrics did not differ significantly between healthy elderly eyes and eyes with iAMD. In contrast, nAMD eyes showed fewer but thicker choroidal vessels and an increased stromal fraction, resulting in sparse, enlarged vessels within expanded connective tissue. This may reflect impaired choroidal perfusion more severely, potentially compromising oxygen and nutrient delivery to the outer retina and contributing to disease progression. In addition, the significant reduction of FVA and FVC in nAMD compared with the other elderly cohorts in our study may suggest more pronounced central choroidal regression in nAMD. However, this interpretation is limited by the fact that foveal choroidal vessel parameters may be substantially influenced by transmission changes caused by the overlying MNV.

Several mechanisms may account for the choroidal vascular alterations. Impaired diffusion across Bruch’s membrane (BM) with reduced availability of VEGF has been proposed as a central contributor to choroidal vascular loss [27, 28]. Severe choriocapillaris loss and vascular rarefaction have been described in conditions characterized by sub-RPE deposit accumulation, such as Sorsby fundus dystrophy [29].

In nAMD, delayed choroidal filling on angiography has similarly been interpreted as evidence of reduced choroidal perfusion, potentially resulting from lipid-rich thickening of BM and impaired VEGF diffusion [30].

Experimental and clinical data further indicate that both the choriocapillaris and larger choroidal vessels are highly VEGF-dependent with progressive vascular regression observed during GA pathogenesis [14].

In this context, progressive thickening of BM with lipid accumulation may create a hydrophobic diffusion barrier, ultimately promoting choroidal vascular rarefaction [30, 31].

Choroidal thinning may be influenced by systemic, particularly cerebral perfusion. An arteriosclerotic contribution to AMD-related choroidal changes has been proposed based on studies demonstrating a close relationship between ocular perfusion and choroidal morphology [32].

Recent clinical data demonstrate improved choroidal and choriocapillar perfusion with increased CT following carotid endarterectomy [33]. Consistently, the diffuse-trickling GA phenotype is associated with choroidal thinning and increased myocardial infarction risk, supporting a link between systemic vascular pathology and choroidal degeneration [34].

Certain limitations of this study should be acknowledged. First, no formal power calculation was performed during study planning. Consequently, the imbalance between groups may have affected statistical power and the interpretation of the results, particularly with regard to non-significant findings. In addition, some eyes classified as healthy elderly or iAMD may eventually progress to nAMD, which limits the strict separation of the cohorts.

Second, only nAMD eyes with evaluable choroidal segmentations were included. Eyes with severe transmission defects or insufficient choroidal visibility, for example due to large MNV lesions, were excluded. Nevertheless, AI-based segmentation of choroidal vessels beneath MNV may remain susceptible to artifacts from overlying pathology. Since systemic vascular conditions may influence choroidal perfusion and such data were not available for the elderly cohorts, this factor may have contributed to the observed differences. Similar limitations apply to axial length, another potential determinant of choroidal thickness, for which data were also not available. Age distributions were comparable between the elderly cohorts, and the sex distribution was similar between the healthy elderly and iAMD groups. The nAMD group contained a higher proportion of female participants another factor potentially influencing the results.

Automated segmentations were visually verified by a trained grader and required only minimal corrections, although manual review may introduce a small degree of observer-dependent variability.

In conclusion, a marked age-related reduction in both vascular and stromal choroidal structures was observed.

This reduction is likely driven by arteriosclerotic alterations affecting systemic and ocular perfusion. While choroidal morphology was comparable between healthy elderly and iAMD eyes, treatment-naive nAMD showed a further disproportionate loss of the vascular component.

The combined effects of reduced systemic perfusion and impaired VEGF diffusion may lead to progressive choroidal hypoperfusion and insufficient metabolic support of the outer retina. Once a critical threshold is reached, potentially reflected by a pronounced reduction in CVI, MNV may arise as a compensatory neo-choriocapillaris [35]. CVI may represent a clinically meaningful biomarker of choroidal perfusion quality. Strategies to preserve or improve choroidal perfusion in iAMD could help prevent progression to nAMD. Future longitudinal studies should investigate whether choroidal vascular rarefaction precedes conversion to nAMD using annotation-derived CVI. 

## Data Availability

The data from this study are available from the corresponding authors upon reasonable request. The code and trained model checkpoints are accessible at the following link: ( https://github.com/TIO-IKIM/AMD_SemSeg ).

## References

[1] Fleckenstein M, Schmitz-Valckenberg S, Chakravarthy U (2024) Age-related macular degeneration: a review. JAMA 331(2):147–157. 10.1001/jama.2023.26074 PMC12935482 38193957

[2] Wong WL, Su X, Li X, Cheung CM, Klein R, Cheng CY et al (2014) Global prevalence of age-related macular degeneration and disease burden projection for 2020 and 2040: a systematic review and meta-analysis. Lancet Glob Health 2(2):e106–e116. 10.1016/S2214-109X(13)70145-1 25104651

[3] Kim J, Lee YJ, Won JY (2021) Molecular mechanisms of retinal pigment epithelium dysfunction in age-related macular degeneration. Int J Mol Sci 22(22). 10.3390/ijms222212298 PMC8624542 34830181

[4] Chirco KR, Sohn EH, Stone EM, Tucker BA, Mullins RF (2017) Structural and molecular changes in the aging choroid: implications for age-related macular degeneration. Eye (Lond) 31(1):10–25. 10.1038/eye.2016.216 PMC5233940 27716746

[5] Spaide RF, Jaffe GJ, Sarraf D, Freund KB, Sadda SR, Staurenghi G et al (2020) Consensus nomenclature for reporting neovascular age-related macular degeneration data: consensus on neovascular age-related macular degeneration nomenclature study group. Ophthalmology 127(5):616–636. 10.1016/j.ophtha.2019.11.004 PMC11559632 31864668

[6] Rosenfeld PJ, Brown DM, Heier JS, Boyer DS, Kaiser PK, Chung CY et al (2006) Ranibizumab for neovascular age-related macular degeneration. N Engl J Med 355(14):1419–1431. 10.1056/NEJMoa054481 17021318

[7] Grunwald JE, Daniel E, Huang J, Ying GS, Maguire MG, Toth CA et al (2014) Risk of geographic atrophy in the comparison of age-related macular degeneration treatments trials. Ophthalmology 121(1):150–161. 10.1016/j.ophtha.2013.08.015 PMC3892560 24084496

[8] Sadda SR, Tuomi LL, Ding B, Fung AE, Hopkins JJ (2018) Macular Atrophy in the HARBOR Study for Neovascular Age-Related Macular Degeneration. Ophthalmology 125(6):878–886. 10.1016/j.ophtha.2017.12.026 29477692

[9] Spooner KL, Fraser-Bell S, Cozzi M, Staurenghi G, Invernizzi A, Monteduro D et al (2020) Macular Atrophy Incidence and Progression in Eyes with Neovascular Age-Related Macular Degeneration Treated with Vascular Endothelial Growth Factor Inhibitors Using a Treat-and-Extend or a Pro Re Nata Regimen: Four-Year Results of the MANEX Study. Ophthalmology 127(12):1663–1673. 10.1016/j.ophtha.2020.06.019 32544561

[10] Gunnemann ML, Ziegler M, Book M, Gunnemann F, Rothaus K, Spital G et al (2024) Is Exudative Neovascular AMD a Chronic Disease? Analysis of Long-term Progression under Anti-VEGF Therapy. Klin Monbl Augenheilkd 241(12):1335–1340. 10.1055/a-2239-6394 38181813

[11] Fisher CR, Ferrington DA (2018) Perspective on AMD Pathobiology: A Bioenergetic Crisis in the RPE. Invest Ophthalmol Vis Sci 59(4):AMD41–AMD7. 10.1167/iovs.18-24289 PMC5989860 30025108

[12] Mammadzada P, Corredoira PM, Andre H (2020) The role of hypoxia-inducible factors in neovascular age-related macular degeneration: a gene therapy perspective. Cell Mol Life Sci 77(5):819–833. 10.1007/s00018-019-03422-9 PMC7058677 31893312

[13] Spaide RF (2009) Age-related choroidal atrophy. Am J Ophthalmol 147(5):801–810. 10.1016/j.ajo.2008.12.010 19232561

[14] Borrelli E, Cappellani F, Pulido JS, Pauleikhoff D, Bhutto IA, McLeod DS et al (2024) The Umbra and the Penumbra: Longitudinal Effects of Geographic Atrophy in AMD on the Outer Choroid by Imaging Analysis and Histopathological Correlation. Invest Ophthalmol Vis Sci 65(14):2. 10.1167/iovs.65.14.2 PMC11614027 39620831

[15] Romano F, Ding X, Yuan M, Vingopoulos F, Garg I, Choi H et al (2024) Progressive choriocapillaris changes on optical coherence tomography angiography correlate with stage progression in AMD. Invest Ophthalmol Vis Sci 65(8):21. 10.1167/iovs.65.8.21 PMC11246100 38990069

[16] Shen M, Zhou H, Lu J, Li J, Jiang X, Trivizki O et al (2023) Choroidal Changes After Anti-VEGF Therapy in AMD Eyes With Different Types of Macular Neovascularization Using Swept-Source OCT Angiography. Invest Ophthalmol Vis Sci 64(13):16. 10.1167/iovs.64.13.16 PMC10573575 37819744

[17] Borrelli E, Mohammed AR, Barresi C, Vupparaboina KK, Viggiano P, Boscia F et al (2025) Capturing the transition from intermediate to neovascular AMD: Longitudinal changes in choroidal volume and choroidal vascularity index. Graefes Arch Clin Exp Ophthalmol 263(5):1289–1296. 10.1007/s00417-025-06767-z PMC12148963 39939555

[18] Sonoda S, Sakamoto T, Yamashita T, Uchino E, Kawano H, Yoshihara N et al (2015) Luminal and stromal areas of choroid determined by binarization method of optical coherence tomographic images. Am J Ophthalmol 159(6):1123–31e1. 10.1016/j.ajo.2015.03.005 25790737

[19] Iovino C, Pellegrini M, Bernabei F, Borrelli E, Sacconi R, Govetto A et al (2020) Choroidal vascularity index: an in-depth analysis of this novel optical coherence tomography parameter. J Clin Med 9(2). 10.3390/jcm9020595 PMC7074450 32098215

[20] Kumar M, Khuu SK, Trinh M, Madigan MC, Agrawal R, Rojas-Carabali W et al (2025) Choroidal vascularity index is independent of ocular and image-based factors in healthy eyes: a systematic review and meta-analysis. Sci Rep 15(1):27782. 10.1038/s41598-025-10384-5 PMC12311116 40739311

[21] Agrawal R, Gupta P, Tan KA, Cheung CM, Wong TY, Cheng CY (2016) Choroidal vascularity index as a measure of vascular status of the choroid: Measurements in healthy eyes from a population-based study. Sci Rep 6:21090. 10.1038/srep21090 PMC4751574 26868048

[22] Nivison-Smith L, Khandelwal N, Tong J, Mahajan S, Kalloniatis M, Agrawal R (2020) Normal aging changes in the choroidal angioarchitecture of the macula. Sci Rep 10(1):10810. 10.1038/s41598-020-67829-2 PMC7331638 32616774

[23] Stasch-Bouws J, Eller-Woywod SM, Schmickler S, Inderfurth J, Hoffmann P, Ohlmeyer C et al (2020) [IVOM quality assurance in Westfalen-Lippe: Structure of quality assurance and results of the pilot study Q-VERA]. Ophthalmologe 117(4):336–342. 10.1007/s00347-019-01030-3 31912271

[24] Heine L, Vahldiek A, Vahldiek B, Horst F, Seibold C, Lever M et al (2025) Three-dimensional quantification of macular oct alterations improves the diagnostic performance of artificial intelligence models. Transl Vis Sci Technol 14(7):8. 10.1167/tvst.14.7.8 PMC12279069 40668050

[25] Vahldiek A, Heine L, Vahldiek B, Schroter J, Wolf JN, Swora M et al (2025) Automated measurement of macular neovascularization lesion size in nAMD using AI segmentation. Graefes Arch Clin Exp Ophthalmol. 10.1007/s00417-025-07007-0 PMC12923400 41247474

[26] Rosa N, Gioia M, Orlando R, De Luca M, D’Aniello E, Fioretto I et al (2024) Impact of brightness on choroidal vascularity index. J Clin Med 13(4). 10.3390/jcm13041020 PMC10889141 38398333

[27] Saint-Geniez M, Kurihara T, Sekiyama E, Maldonado AE, D’Amore PA (2009) An essential role for RPE-derived soluble VEGF in the maintenance of the choriocapillaris. Proc Natl Acad Sci U S A 106(44):18751–18756. 10.1073/pnas.0905010106 PMC2774033 19841260

[28] Moore DJ, Hussain AA, Marshall J (1995) Age-related variation in the hydraulic conductivity of Bruch’s membrane. Invest Ophthalmol Vis Sci 36(7):1290–1297 7775106

[29] Capon MR, Marshall J, Krafft JI, Alexander RA, Hiscott PS, Bird AC (1989) Sorsby’s fundus dystrophy. A light and electron microscopic study. Ophthalmology 96(12):1769–1777. 10.1016/s0161-6420(89)32664-9 2482957

[30] Pauleikhoff D, Chen JC, Chisholm IH, Bird AC (1990) Choroidal perfusion abnormality with age-related Bruch’s membrane change. Am J Ophthalmol 109(2):211–217. 10.1016/s0002-9394(14)75989-6 2301534

[31] Clark SJ, Curcio C, Dick AD, Doyle S, Edwards M, Flores-Bellver M et al (2025) Breaking bruch’s: how changes in bruch’s membrane influence retinal homeostasis. Exp Eye Res 255:110343. 10.1016/j.exer.2025.110343 40107443

[32] Friedman E, Krupsky S, Lane AM, Oak SS, Friedman ES, Egan K et al (1995) Ocular blood flow velocity in age-related macular degeneration. Ophthalmology 102(4):640–646. 10.1016/s0161-6420(95)30974-8 7724181

[33] Berni A, Zhang Y, Wenting SZ, Noam N, Rabinovitch D, Yousif BS et al (2025) Long-term impact of carotid endarterectomy on choroidal and choriocapillaris perfusion: the INFLATE study. Ophthalmol Sci 5(2):100651. 10.1016/j.xops.2024.100651 PMC11754506 39850542

[34] Fleckenstein M, Schmitz-Valckenberg S, Lindner M, Bezatis A, Becker E, Fimmers R et al (2014) The diffuse-trickling fundus autofluorescence phenotype in geographic atrophy. Invest Ophthalmol Vis Sci 55(5):2911–2920. 10.1167/iovs.13-13409 24699379

[35] Corvi F, Bacci T, Corradetti G, Staurenghi G, Sarraf D, Freund KB et al (2023) Characterisation of the vascular anterior surface of type 1 macular neovascularisation after anti-VEGF therapy. Br J Ophthalmol 107(9):1336–1343. 10.1136/bjophthalmol-2021-320047 35537801

